# 4-Chloro-3-nitro­benzamide

**DOI:** 10.1107/S1600536808041342

**Published:** 2008-12-10

**Authors:** Bo-Nian Liu, Shi-Gui Tang, Hao-Yuan Li, Cheng Guo

**Affiliations:** a College of Science, Nanjing University of Technology, Xinmofan Road No. 5, Nanjing 210009, People’s Republic of China; b College of Life Sciences and Pharmaceutical Engineering, Nanjing University of Technology, Nanjing 210009, People’s Republic of China

## Abstract

In the crystal of the title compound, C_7_H_5_ClN_2_O_3_, the molecules are linked by N—H⋯O and C—H⋯O hydrogen bonds. The π–π contact between the benzene rings, [centroid–centroid distance = 3.803 (3) Å] may further stabilize the structure.

## Related literature

For a related structure, see: Sun *et al.* (2006 ▶). For bond-length data, see: Allen *et al.* (1987 ▶).
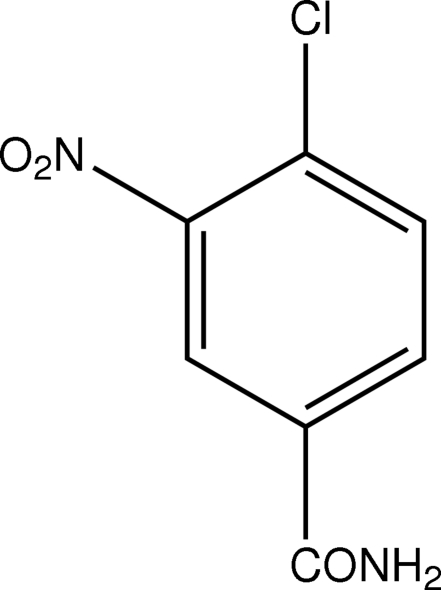


## Experimental

### 

#### Crystal data

C_7_H_5_ClN_2_O_3_
*M* *_r_* = 200.58
Monoclinic, 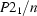

*a* = 8.8490 (18) Å
*b* = 7.5470 (15) Å
*c* = 12.374 (3) Å
β = 101.18 (3)°
*V* = 810.7 (3) Å^3^
*Z* = 4
Mo *K*α radiation
μ = 0.44 mm^−1^
*T* = 294 (2) K
0.30 × 0.20 × 0.10 mm

#### Data collection

Enraf–Nonius CAD-4 diffractometer
Absorption correction: ψ scan (North *et al.*, 1968 ▶) *T*_min_ = 0.879, *T*_max_ = 0.957
1555 measured reflections
1459 independent reflections
1085 reflections with *I* > 2σ(*I*)
*R*_int_ = 0.061
3 standard reflections frequency: 120 min intensity decay: none

#### Refinement

*R*[*F* ^2^ > 2σ(*F* ^2^)] = 0.078
*wR*(*F* ^2^) = 0.198
*S* = 1.01
1459 reflections
112 parameters
H-atom parameters constrained
Δρ_max_ = 0.41 e Å^−3^
Δρ_min_ = −0.51 e Å^−3^

### 

Data collection: *CAD-4 Software* (Enraf–Nonius, 1989 ▶); cell refinement: *CAD-4 Software*; data reduction: *XCAD4* (Harms & Wocadlo, 1995 ▶); program(s) used to solve structure: *SHELXS97* (Sheldrick, 2008 ▶); program(s) used to refine structure: *SHELXL97* (Sheldrick, 2008 ▶); molecular graphics: *ORTEP-3 for Windows* (Farrugia, 1997 ▶) and *PLATON* (Spek, 2003 ▶); software used to prepare material for publication: *SHELXL97* and *PLATON*.

## Supplementary Material

Crystal structure: contains datablocks global, I. DOI: 10.1107/S1600536808041342/hk2596sup1.cif

Structure factors: contains datablocks I. DOI: 10.1107/S1600536808041342/hk2596Isup2.hkl

Additional supplementary materials:  crystallographic information; 3D view; checkCIF report

## Figures and Tables

**Table 1:** Hydrogen-bond geometry (Å, °)

*D*—H⋯*A*	*D*—H	H⋯*A*	*D*⋯*A*	*D*—H⋯*A*
N2—H2*B*⋯O3^i^	0.86	2.10	2.958 (6)	177
N2—H2*C*⋯O2^ii^	0.86	2.26	3.067 (6)	155
C2—H2*A*⋯O3^iii^	0.93	2.42	3.331 (6)	166

Symmetry codes: (i) 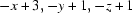; (ii) 
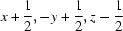
; (iii) 
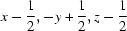
.

**Fig. 1.**
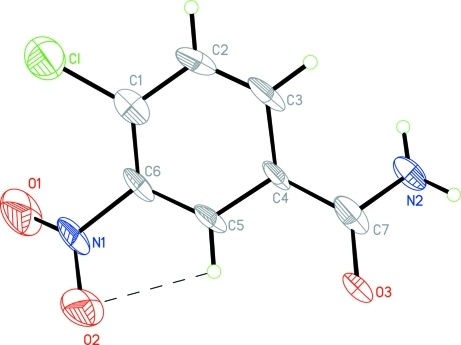
The molecular structure of the title molecule, with the atom-numbering scheme. Displacement ellipsoids are drawn at the 30% probability level. Hydrogen bond is shown as dashed line.

**Fig. 2.**
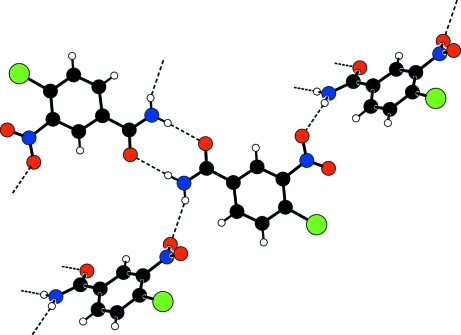
A partial packing diagram of the title compound. Hydrogen bonds are shown as dashed lines.

## supplementary crystallographic information

### Comment

Some derivatives of pyridine are important chemical materials. We report herein the crystal structure of the title compound.

In the molecule of the title compound (Fig 1), the bond lengths (Allen *et al.*,  1987) and angles are within normal ranges. Ring A (C1-C6) is, of course, planar. Atoms Cl, N1 and C7 are 0.021 (3), 0.029 (3) and -0.001 (3) Å away from the plane of the benzene ring. The intramolecular C-H···O hydrogen bond results in the formation of a five-membered ring B (O2/N1/C5/C6/H5A), having envelope conformation with O2 atom displaced by 0.278 (3) Å from the plane of the other ring atoms.

In the crystal structure, intermolecular N-H···O and C-H···O hydrogen bonds (Table 1) link the molecules (Fig. 2), in which they may be effective in the stabilization of the structure. The π-π contact between the benzene rings, Cg1—Cg1^i^ [symmetry code: (i) -x, -y, 1 - z, where Cg1 is centroid of the ring A (C1-C6)] may further stabilize the structure, with centroid-centroid distance of 3.803 (3) Å.

### Experimental

For the preparation of the title compound, 4-chloro-3-nitrobenzoic acid (60.3 g, 0.32 mol) was suspended in thionyl chloride (180 ml) and heated at reflux for 5 h, then concentrated in vacuum as far as possible, the oily substance obtained. Added ice ammonia water (300 ml) to the oil, cooling to room temperature, a precipitate formed, which was collected by filtration and washed with water. Pure title compound was obtained by crystallizing from methanol (Sun *et al.*, 2006). Crystals suitable for X-ray analysis were obtained by slow evaporation of a methanol solution.

### Refinement

H atoms were positioned geometrically, with N-H = 0.86 (for NH_2_) and C-H = 0.93 Å for aromatic H and constrained to ride on their parent atoms, with U_iso_(H) = 1.2U_eq_(C,N).

### Crystal data

**Table:**

C_7_H_5_ClN_2_O_3_	*F*(000) = 408
*M**_r_* = 200.58	*D*_x_ = 1.643 Mg m^−^^3^
Monoclinic, *P*2_1_/*n*	Mo *K*α radiation, λ = 0.71073 Å
Hall symbol: -P 2yn	Cell parameters from 25 reflections
*a* = 8.8490 (18) Å	θ = 10–13°
*b* = 7.5470 (15) Å	µ = 0.44 mm^−^^1^
*c* = 12.374 (3) Å	*T* = 294 K
β = 101.18 (3)°	Block, colorless
*V* = 810.7 (3) Å^3^	0.30 × 0.20 × 0.10 mm
*Z* = 4	

### Data collection

**Table:**

Enraf–Nonius CAD-4 diffractometer	1085 reflections with *I* > 2σ(*I*)
Radiation source: fine-focus sealed tube	*R*_int_ = 0.061
graphite	θ_max_ = 25.3°, θ_min_ = 2.6°
ω/2θ scans	*h* = −10→10
Absorption correction: ψ scan (North *et al.*, 1968)	*k* = 0→9
*T*_min_ = 0.879, *T*_max_ = 0.957	*l* = 0→14
1555 measured reflections	3 standard reflections every 120 min
1459 independent reflections	intensity decay: none

### Refinement

**Table:**

Refinement on *F*^2^	Secondary atom site location: difference Fourier map
Least-squares matrix: full	Hydrogen site location: inferred from neighbouring sites
*R*[*F*^2^ > 2σ(*F*^2^)] = 0.078	H-atom parameters constrained
*wR*(*F*^2^) = 0.198	*w* = 1/[σ^2^(*F*_o_^2^) + (0.060*P*)^2^ + 4.5*P*] where *P* = (*F*_o_^2^ + 2*F*_c_^2^)/3
*S* = 1.01	(Δ/σ)_max_ < 0.001
1459 reflections	Δρ_max_ = 0.41 e Å^−^^3^
112 parameters	Δρ_min_ = −0.50 e Å^−^^3^
Primary atom site location: structure-invariant direct methods	

### Special details

**Table:**

Geometry. All e.s.d.'s (except the e.s.d. in the dihedral angle between two l.s. planes) are estimated using the full covariance matrix. The cell e.s.d.'s are taken into account individually in the estimation of e.s.d.'s in distances, angles and torsion angles; correlations between e.s.d.'s in cell parameters are only used when they are defined by crystal symmetry. An approximate (isotropic) treatment of cell e.s.d.'s is used for estimating e.s.d.'s involving l.s. planes.
Refinement. Refinement of *F*^2^ against ALL reflections. The weighted *R*-factor *wR* and goodness of fit *S* are based on *F*^2^, conventional *R*-factors *R* are based on *F*, with *F* set to zero for negative *F*^2^. The threshold expression of *F*^2^ > σ(*F*^2^) is used only for calculating *R*-factors(gt) *etc*. and is not relevant to the choice of reflections for refinement. *R*-factors based on *F*^2^ are statistically about twice as large as those based on *F*, and *R*- factors based on ALL data will be even larger.

### Fractional atomic coordinates and isotropic or equivalent isotropic displacement parameters (Å^2^)

**Table:**

	*x*	*y*	*z*	*U*_iso_*/*U*_eq_	
Cl	0.65545 (17)	0.0416 (2)	0.36001 (13)	0.0618 (5)	
O1	0.7053 (5)	0.1130 (7)	0.5888 (4)	0.0749 (14)	
O2	0.8610 (5)	0.3068 (6)	0.6677 (3)	0.0554 (11)	
O3	1.3178 (4)	0.4669 (5)	0.5500 (2)	0.0390 (9)	
N1	0.8103 (5)	0.2076 (5)	0.5896 (3)	0.0385 (10)	
N2	1.3809 (5)	0.3507 (7)	0.3985 (4)	0.0502 (12)	
H2B	1.4701	0.4002	0.4120	0.060*	
H2C	1.3546	0.2856	0.3409	0.060*	
C1	0.8373 (6)	0.1359 (7)	0.3967 (4)	0.0378 (11)	
C2	0.9221 (6)	0.1454 (7)	0.3143 (4)	0.0401 (12)	
H2A	0.8819	0.1004	0.2448	0.048*	
C3	1.0637 (6)	0.2201 (7)	0.3346 (4)	0.0424 (12)	
H3A	1.1188	0.2260	0.2779	0.051*	
C4	1.1312 (5)	0.2902 (6)	0.4397 (3)	0.0295 (10)	
C5	1.0412 (5)	0.2840 (6)	0.5196 (3)	0.0316 (10)	
H5A	1.0790	0.3319	0.5888	0.038*	
C6	0.8958 (5)	0.2076 (6)	0.4985 (4)	0.0308 (10)	
C7	1.2837 (6)	0.3746 (7)	0.4668 (4)	0.0435 (10)	

### Atomic displacement parameters (Å^2^)

**Table:**

	*U*^11^	*U*^22^	*U*^33^	*U*^12^	*U*^13^	*U*^23^
Cl	0.0584 (9)	0.0679 (10)	0.0626 (10)	−0.0058 (7)	0.0208 (7)	−0.0114 (8)
O1	0.079 (3)	0.088 (3)	0.074 (3)	−0.027 (3)	0.057 (3)	−0.007 (3)
O2	0.072 (3)	0.068 (3)	0.035 (2)	−0.016 (2)	0.0311 (18)	−0.007 (2)
O3	0.0454 (18)	0.052 (2)	0.0267 (16)	−0.0091 (16)	0.0254 (14)	−0.0091 (15)
N1	0.054 (2)	0.038 (2)	0.036 (2)	0.000 (2)	0.0375 (19)	0.0044 (19)
N2	0.051 (2)	0.070 (3)	0.042 (2)	−0.007 (2)	0.038 (2)	−0.018 (2)
C1	0.049 (3)	0.035 (3)	0.035 (2)	0.008 (2)	0.020 (2)	0.004 (2)
C2	0.063 (3)	0.042 (3)	0.019 (2)	0.001 (2)	0.018 (2)	−0.004 (2)
C3	0.068 (3)	0.044 (3)	0.024 (2)	0.002 (2)	0.031 (2)	0.002 (2)
C4	0.043 (2)	0.030 (2)	0.022 (2)	−0.0015 (19)	0.0245 (18)	−0.0009 (18)
C5	0.050 (3)	0.032 (2)	0.020 (2)	0.002 (2)	0.0234 (18)	−0.0002 (18)
C6	0.046 (2)	0.028 (2)	0.027 (2)	0.0066 (19)	0.0275 (19)	0.0056 (18)
C7	0.061 (2)	0.038 (2)	0.038 (2)	0.009 (19)	0.034 (19)	0.006 (18)

### Geometric parameters (Å, °)

**Table:**

Cl—C1	1.737 (5)	C1—C6	1.377 (7)
O3—C7	1.231 (6)	C2—C3	1.352 (7)
N1—O1	1.170 (6)	C2—H2A	0.9300
N1—O2	1.236 (5)	C3—C4	1.424 (7)
N1—C6	1.474 (5)	C3—H3A	0.9300
N2—C7	1.329 (6)	C4—C5	1.386 (6)
N2—H2B	0.8600	C4—C7	1.471 (7)
N2—H2C	0.8600	C5—C6	1.388 (7)
C1—C2	1.380 (6)	C5—H5A	0.9300
			
O1—N1—O2	122.9 (4)	C4—C3—H3A	118.9
O1—N1—C6	121.2 (4)	C3—C4—C7	124.9 (4)
O2—N1—C6	115.8 (4)	C5—C4—C3	116.2 (4)
C7—N2—H2B	120.0	C5—C4—C7	118.8 (4)
C7—N2—H2C	120.0	C4—C5—C6	121.3 (4)
H2B—N2—H2C	120.0	C4—C5—H5A	119.3
C2—C1—Cl	115.9 (4)	C6—C5—H5A	119.3
C6—C1—Cl	124.4 (4)	C1—C6—C5	120.4 (4)
C6—C1—C2	119.6 (5)	C1—C6—N1	122.8 (4)
C1—C2—H2A	119.9	C5—C6—N1	116.8 (4)
C3—C2—C1	120.1 (5)	O3—C7—N2	121.7 (5)
C3—C2—H2A	119.9	O3—C7—C4	120.0 (4)
C2—C3—C4	122.3 (4)	N2—C7—C4	118.3 (5)
C2—C3—H3A	118.9		
			
O1—N1—C6—C1	17.6 (7)	C2—C3—C4—C5	−2.5 (7)
O2—N1—C6—C1	−165.6 (5)	C2—C3—C4—C7	−179.3 (5)
O1—N1—C6—C5	−162.2 (5)	C3—C4—C5—C6	2.3 (7)
O2—N1—C6—C5	14.6 (6)	C7—C4—C5—C6	179.3 (4)
C6—C1—C2—C3	2.0 (8)	C5—C4—C7—O3	−13.8 (7)
Cl—C1—C2—C3	178.6 (4)	C3—C4—C7—O3	163.0 (5)
C2—C1—C6—C5	−2.2 (7)	C5—C4—C7—N2	167.3 (5)
Cl—C1—C6—C5	−178.5 (4)	C3—C4—C7—N2	−15.9 (8)
C2—C1—C6—N1	178.1 (4)	C4—C5—C6—C1	0.0 (7)
Cl—C1—C6—N1	1.8 (7)	C4—C5—C6—N1	179.7 (4)
C1—C2—C3—C4	0.4 (8)		

### Hydrogen-bond geometry (Å, °)

**Table:**

*D*—H···*A*	*D*—H	H···*A*	*D*···*A*	*D*—H···*A*
N2—H2B···O3^i^	0.86	2.10	2.958 (6)	177
N2—H2C···O2^ii^	0.86	2.26	3.067 (6)	155
C2—H2A···O3^iii^	0.93	2.42	3.331 (6)	166
C5—H5A···O2	0.93	2.33	2.658 (6)	100

Symmetry codes: (i) −*x*+3, −*y*+1, −*z*+1; (ii) *x*+1/2, −*y*+1/2, *z*−1/2; (iii) *x*−1/2, −*y*+1/2, *z*−1/2.

## References

[bb1] Allen, F. H., Kennard, O., Watson, D. G., Brammer, L., Orpen, A. G. & Taylor, R. (1987). * J. Chem. Soc. Perkin Trans. 2*, pp. S1–19.

[bb2] Enraf–Nonius (1989). *CAD-4 Software* Enraf–Nonius, Delft. The Netherlands.

[bb3] Farrugia, L. J. (1997). *J. Appl. Cryst.* **30**, 565.

[bb4] Harms, K. & Wocadlo, S. (1995). *XCAD4* University of Marburg, Germany.

[bb5] North, A. C. T., Phillips, D. C. & Mathews, F. S. (1968). *Acta Cryst.* A**24**, 351–359.

[bb6] Sheldrick, G. M. (2008). *Acta Cryst.* A**64**, 112–122. 10.1107/S0108767307043930 18156677

[bb7] Spek, A. L. (2003). *J. Appl. Cryst.* **36**, 7–13.

[bb8] Sun, Y. W. & Wang, J. W. (2006). *Hua Xue Shi Ji*, **28**, 124–125.

